# Luminescence Thermometry on the Route of the Mobile‐Based Internet of Things (IoT): How Smart QR Codes Make It Real

**DOI:** 10.1002/advs.201900950

**Published:** 2019-08-09

**Authors:** João F. C. B. Ramalho, Sandra F. H. Correia, Lianshe Fu, Lara L. F. António, Carlos D. S. Brites, Paulo S. André, Rute A. S. Ferreira, Luís D. Carlos

**Affiliations:** ^1^ Department of Physics and CICECO – Aveiro Institute of Materials University of Aveiro 3810‐193 Aveiro Portugal; ^2^ Department of Electronics, Telecommunications and Informatics Instituto de Telecomunicações University of Aveiro 3810‐193 Aveiro Portugal; ^3^ Department of Electric and Computer Engineering and Instituto de Telecomunicações Instituto Superior Técnico Universidade de Lisboa 1049‐001 Lisbon Portugal

**Keywords:** color multiplexing, lanthanide ions, mobile apps, molecular thermometry, organic–inorganic hybrids, Quick Response (QR) codes

## Abstract

Quick Response (QR) codes are a gateway to the Internet of things (IoT) due to the growing use of smartphones/mobile devices and its properties like fast and easy reading, capacity to store more information than that found in conventional codes, and versatility associated to the rapid and simplified access to information. Challenges encompass the enhancement of storage capacity limits and the evolution to a smart label for mobile devices decryption applications. Organic–inorganic hybrids with europium (Eu^3+^) and terbium (Tb^3+^) ions are processed as luminescent QR codes that are able to simultaneously double the storage capacity and sense temperature in real time using a photo taken with the charge‐coupled device of a smartphone. The methodology based on the intensity of the red and green pixels of the photo yields a maximum relative sensitivity and minimum temperature uncertainty of the QR code sensor (293 K) of 5.14% · K^−1^ and 0.194 K, respectively. As an added benefit, the intriguing performance results from energy transfer involving the thermal coupling between the Tb^3+^‐excited level (^5^D_4_) and the low‐lying triplet states of organic ligands, being the first example of an intramolecular primary thermometer. A mobile app is developed to materialize the concept of temperature reading through luminescent QR codes.

## 1 Introduction

The prominent advancement in mobile technologies has allowed an ever‐closer approach between the user and the access to information, in a more intuitive and simplified way. One of the mechanisms able to promote this approach is based on the use of Quick Response (QR) codes. A QR code consists of a 2D matrix composed of white (inactive) and black (active) square modules capable of encoding information and was first presented in 1994 by the Japanese company Denso Wave Incorporated. Since then, QR codes have been sparking interest and visibility among markets and users. The ability to correct errors in damaged zones, the variety of supported coding languages (e.g., numeric, alphanumeric, kanji, kana), the low cost and easy production and the high storage capacity are greater advantages of QR codes, compared with other labels (linear barcodes, RFID, NFC tags). Therefore, it is not surprising that QR codes are now employed in distinct areas of daily life and with different extents, such as anti‐counterfeiting and luminescent on‐demand tags,^1^, ^2^, ^3^ industry 4.0,^4^ security (authentication of documents for legal, institutional, or other equivalent purposes),^5^ food science and nutrition,^6^ and Internet of Things (IoT).^7^

With such broad spectrum of applications, in recent years the emphasis has been placed on optimizing and developing the capabilities of QR codes, in order to mitigate their weaknesses. Main challenges encompass the increase of the information storage capacity and the possibility of adding new properties, yielding to active smart labels. Concerning the increase in storage capacity, several methods have been settled.^8^, ^9^, ^10^ Among those, we highlight color multiplexing methods for which the storage capacity can be enhanced with respect to that of a black/white QR code (e.g., eight color codes, obtained with the color multiplexing of three base codes, can triple the data storage),^10^ adding the extra attractive visual effect produced as QR codes become visually more appealing. When luminescent materials are used as inks to print or coat QR codes^1^, ^11^ novel functionalities may be expected by exploring the sensing characteristics associated to color variation with physical parameters, such as temperature. Organic–inorganic hybrids doped with trivalent lanthanide (Ln^3+^) ions^12^ combine the pure and tunable emission of the metal centers with the processing flexibility of hybrid materials, offering the appropriated properties to design luminescent QR codes.^9^ Moreover, several molecular luminescent thermometers based on the temperature dependence of the emission spectra of Ln^3+^ ions embedding into organic–inorganic hybrids have been reported in the past decade.^13^, ^14^, ^15^

Recently, smartphone‐based apparatus in which smartphones are modified with additional components have been used to read luminescent parameters (intensity and lifetime detection)^2^, ^16^, ^17^ and to generate persistent luminescence, through their white‐emitting LEDs coupled to the smartphones' charged‐coupled device (CCD) camera.^17^, ^18^ Fewer examples, however, refer to the combination of luminescent QR codes with smartphones in which although the emission color is quantified applicability is directed to objects authentication (anti‐counterfeiting).^2^, ^19^

In a step forward toward the popularization of luminescent smart QR codes in IoT, here we demonstrate how photographs of luminescent QR codes recorded by a smartphone are used to measure in real‐time the absolute temperature^20^ in the 283–317 K interval with a relative thermal sensitivity (5.14%·K^−1^) and a temperature resolution (0.194 K) better than those typical of electrical counterparts. Since the decoding tool and temperature sensing require only a CCD camera the mobile phone is used in its original configuration. The thermal dependence of the QR code color is quantified by the RGB coordinates, whose G/R ratio is used as the thermometric parameter. Moreover, and as an added benefit, the luminescence thermometer based on the G/R ratio is the first example of a primary intramolecular thermometer allowing to predict the temperature through a well‐defined equation of state prior to any time‐consuming thermal calibration. Luminescence thermometry having become very popular since 2010, particularly due to its enormous potential in micro and nanofluidics, micro and nanoelectronics, photonics, and nanomedicine,^21^ and its connection to QR codes will open exciting horizons on mobile‐based IoT applications.

## 2 Results and Discussion

### 2.1 Luminescent QR Codes

**Figure 1:**
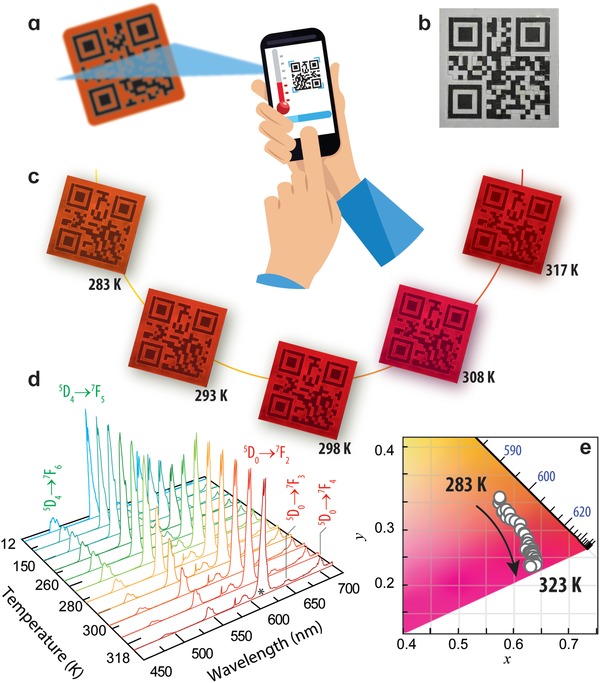
Thermal dependence of the color of the luminescent QR codes. a) Schematic representation of the temperature sensing and message decoding using a smartphone to read the luminescent QR codes. b) Photograph of a multiplexed black/white and luminescent QR code (encoding the message “SMART LABELLING” under daylight. c) Photograph of the luminescent QR codes (encoding the message “INST. DE TELECOMUNICACOES”) under UV illumination at 254 nm for different temperatures (283–317 K). d) Emission spectra under 270 nm excitation recorded between 12 and 318 K. e) Corresponding 1931 CIE emission color coordinates.

**Figure** **1** shows a dU6EuTb‐based luminescent QR code at distinct temperature values, revealing different emission color coordinates, as illustrated in the emission spectra of Figure 1d and in the corresponding 1931 CIE diagram in Figure 1e. The emission is ascribed to the intra‐4f^6^ (Eu^3+^) and intra‐4f^8^ (Tb^3+^) transitions, whose relative intensity is thermal sensitive. As the temperature is raised from 283 to 323 K, the relative intensity of the ^5^D_4_ → ^7^F_6–3_ transitions decreases, whereas that of the ^5^D_0_ → ^7^F_0–4_ transitions remains nearly constant. Consequently, the emission color coordinate deviates from the orange (0.578, 0.356) to the red (0.636, 0.234) spectral regions, Figure 1e.

**Figure 2:**
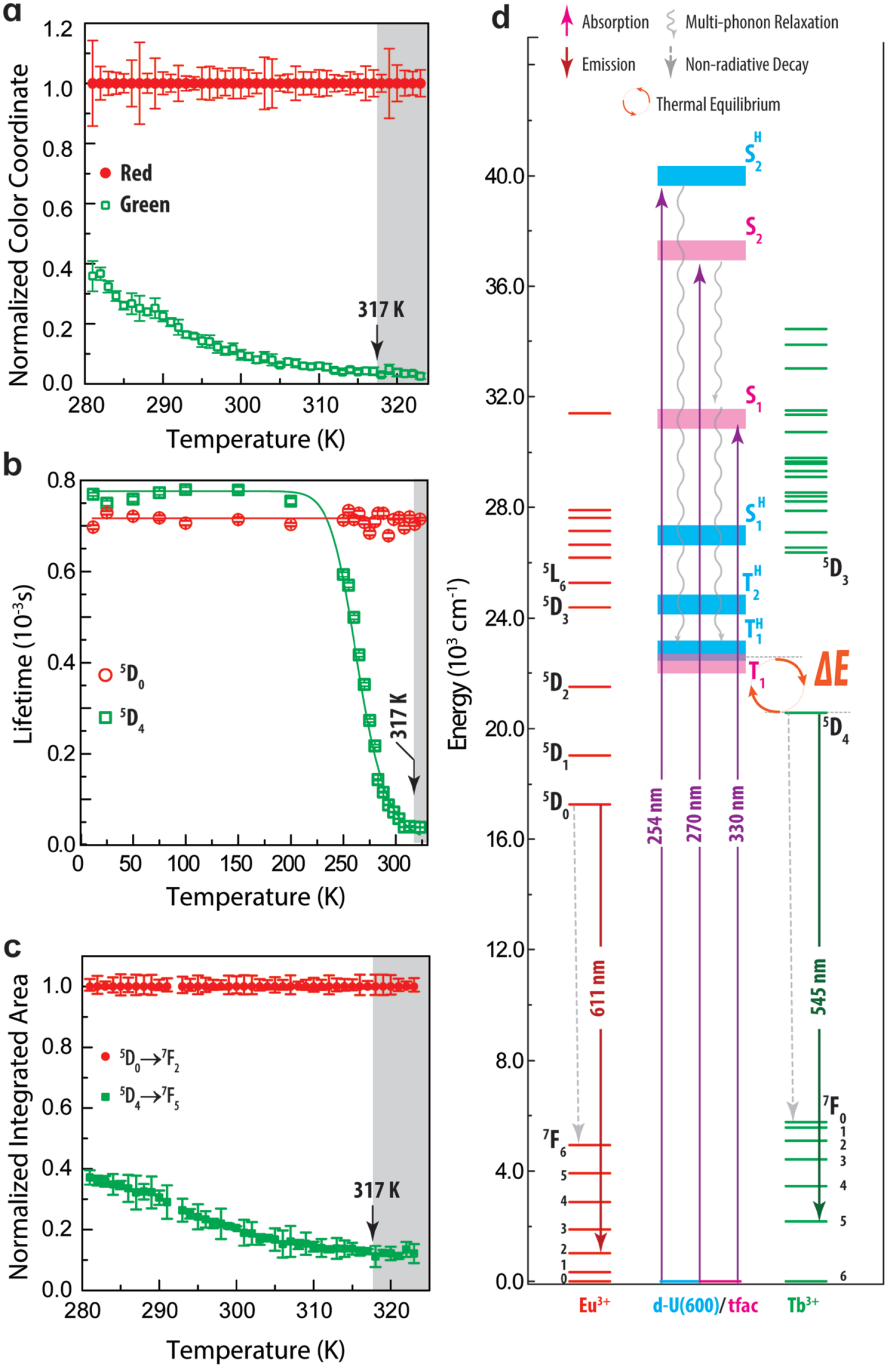
a) Normalized red and green color coordinates variation with temperature (283–323 K) calculated from the photographic records of the luminescent QR code at different temperatures. Temperature dependence of the b) ^5^D_0_ and ^5^D_4_ lifetime values (excitation at 330 nm) and c) integrated areas of the ^5^D_0_ → ^7^F_2_ (*I*_Eu_) and of the ^5^D_4_ → ^7^F_5_ (*I*_Tb_) transitions (excitation at 365 nm). The shadowed areas in a–c mark the region *T* > 317 K in which the G coordinate, ^5^D_4_ lifetime or ^5^D_4_ → ^7^F_5_ integrated area, respectively, change within the error values, and thus the thermometers are out of their operating range. d) Partial energy diagram illustrating potential energy transfer processes in the dU6EuTb, where Δ*E* = 3534 ± 218 cm^−1^.

This color variation with temperature was also quantified in the RBG color space calculated from photographic records of the luminescent QR codes in the same temperature range, **Figure** **2**a, taking advantage of the ability of the smartphone CCD camera that simultaneously allows the QR code reading (decryption) and the photo acquisition for further processing and temperature sensing, through the quantification of the RGB color coordinates of the image avoiding the need of a spectrometer to record emission spectra.

The digital images of the QR codes were split into the projections to the three orthogonal bases, one for each primary color (R, G, and B). For each projection, an intensity histogram describing the number of pixels as functions of the intensity is obtained and modelled with a Gaussian probability density function (pdf), defined by mean value (*µ*) and a color noise variance (σ^2^) (details in Supporting Information).^14^ Figure S4 in the Supporting Information presents the histograms for the R, G and B coordinates, as function of temperature, demonstrating the presence of two distinct peaks in the R histogram, one centered at intensity ≈7, with no noticeable temperature dependence, and another one at intensity ≈110 decreasing as the temperature is raised. In the case of the G histogram, two peaks are discerned at intensities of ≈3 and ≈38. As the temperature is increased, the intensity of the later peak decreases overlapping the low‐intensity one for *T* > 310 K. The B related histogram exhibits two peaks (0 and 6 intensity) both stable with temperature and, thus, negligible in the present context.

Based on this, the split procedure of both QR codes can be made solely recurring to the R histogram, by applying a “maximum likehood” decision criterion (Figure S5, Supporting Information), minimizing the associated error for an optimal decision level. This criterion is used as a threshold value to separate the modules corresponding to the luminescent QR code from the modules of the black/white QR code. The black/white QR code is described by the black color of the active modules and the luminescent QR codes (taken at different temperatures) are well described only by two color coordinates (R and G) for the active modules. After separating both QR codes, it is possible to calculate the RGB coordinates associated to the luminescent material, applying the same pdf fitting (Figure S5, Supporting Information). As the RGB is a 3D orthogonal space, while describing the color of an image it is predictable the formation of *n* clusters, composed by pixels with similar coordinate values, resulting from the noise stochastic process. Therefore, the image composed by pixels with a broad variety of RGB values is transformed, by a simple function, into an image with well‐defined RGB values, described by the centroid point of each cluster, that best identify the exhibited color of luminescent material at each temperature, with an error equals to square root of the color noise variance.^9^, ^10^

In this work we focus on the red and green contributions, as they have a variation with temperature (Figure 1c) and arise from the emission color typical of both lanthanides ions (red and green spectral regions for Eu^3+^ and Tb^3+^, respectively). As illustrated in Figure 2a, the thermal dependence of the normalized R and G coordinates follows the intra‐4^6^ and intra‐4f^8^ thermal dependence, respectively. In particular, while the intensity of the normalized R coordinates remains approximately constant (inside the error margins) between 280 and 325 k, the normalized G coordinates intensity decreases until 317 K, from which a minor variation (within the corresponding uncertainty) is noted. As the R and G intensity becomes nearly constant above 317 K, the thermometer operating range will be restricted to 280–317 K.

To determine the activation range of the QR codes emission, the excitation spectra were selectively monitored within the Eu^3+^ (^5^D_0_ → ^7^F_2_ line at 611 nm) and Tb^3+^ (^5^D_4_ → ^7^F_5_ line at 544 nm) emissions (Figure 2; Figure S6, Supporting Information). Selective excitation spectrum is assured as no spectral overlap occur between the Eu^3+^ and Tb^3+^ transitions nor with the blue intrinsic diureasil emission^22^ (Figure S7a, Supporting Information). The excitation spectra are dominated by a broad band in the UV/blue ascribed to the ligands' singlet excited states.^23^ The negligible intensity of the intra‐4f lines points out that the Ln^3+^ excited states are mainly populated through ligands sensitization. Moreover, the nonobservation of Tb^3+^ transitions in the excitation spectrum selectively monitored within the ^5^D_0_ → ^7^F_2_ transition, supports the absence of Tb^3+^‐to‐Eu^3+^ energy transfer, in accord to the larger number of Tb^3+^ ions compared to those of Eu^3+^ in the binuclear complex, as pointed out by the 1:3 ratio between the Eu^3+^:Tb^3+^ ions (relative concentration of 25% and 75%, respectively, in the dU6EuTb). In that case, the probability of Eu^3+^ and Tb^3+^ ions to be closely located is low, increasing only at larger Eu^3+^:Tb^3+^ ratios. The same conclusion is obtained by the analysis of the low temperature excitation spectra (Figure S6, Supporting Information) that are analogous to those acquired at room temperature. The absence of efficient Tb^3+^‐to‐Eu^3+^ energy transfer is further experimentally supported by i) the ^5^D_0_ lifetime value is independent of the excitation wavelength (Figure S8a, Supporting Information) and of the temperature (Figure 2b); ii) the ^5^D_0_ decay curves do not reveal a rise time typical of Tb^3+^‐to‐Eu^3+^ energy transfer; iii) the ^5^D_4_ emission decay curves overlap up to 200 K which is not compatible with Tb^3+^‐to‐Eu^3+^ energy transfer that is thermally activated at much lower temperature values accordingly to an energy difference around 400 cm^−1^ between the ^5^D_4_ and the ^5^D_2_ levels (main Tb^3+^‐to‐Eu^3+^ energy transfer path); and iv) the emission spectrum excited under selective direct intra‐4f^8^ level (488 nm) reveals only the hybrid host intrinsic emission and no sign of the Eu^3+^‐related ones is detected (Figure S7, Supporting Information).

As a direct consequence of this ligands‐sensitization, the absolute emission quantum yield is 0.13 ± 0.01 under UV excitation. This ensures that light emission is easily discerned under cost‐effective low‐power excitation sources, as light emitting diodes, which is a relevant property envisaging molecular thermometry using the analysis of the QR codes photographic records, as detailed below.

The mechanism behind the emission color dependence on the temperature was studied through the selective acquisition of the ^5^D_0_ (Eu^3+^) and ^5^D_4_ (Tb^3+^) emission decay curves under different UV excitation wavelengths (255–330 nm, Figure 2; Figure S8, Supporting Information). The ^5^D_0_ and ^5^D_4_ decays are independent of the excitation wavelength, yielding analogous lifetime values, as expected since despite distinct excitation wavelengths are used, they involve essentially analogous ligands‐to‐Ln^3+^ energy transfer.^24^ Therefore, the thermal dependence of the decay rates for each state was studied under 330 nm excitation (Figure 2b; FigureS9, Supporting Information). All the decay curves were well‐modeled by a single exponential decay function yielding to the ^5^D_0_ and ^5^D_4_ lifetime values presented in Figure 2b. Whereas the ^5^D_0_ lifetime is temperature independent within the experimental error, that of ^5^D_4_ markedly decreases (≈6×) between 180 and 317 K. The τ(*T*) dependence (Equation (1)) is rationalized on the basis of the classical Mott‐Seitz model that considers the competition between radiative and nonradiative transitions of an emitting level as

(1) $$\tau \left(T\right)\;\;=\;\;\frac{\tau_{0}}{1\;\;+\;\;\alpha \exp\left(-\frac{\Delta E}{k_{B}T}\right)}$$

where τ_0_ is the decay time at the limit *T* → 0 K (also known as the radiative decay time), α is the ratio between the radiative and the nonradiative transfer rates and Δ*E* is the activation energy for the depopulation of the emitting level.^25^ From the best fit of Equation (1) to the experimental ^5^D_4_ lifetime values, results Δ*E* = 3534 ± 218 cm^−1^, α = (2.28 ± 0.26) × 10^8^ and τ_0_ = (0.776 ± 0.020) × 10^−3^ s. We note that τ_0_ value resembles that measured at 14 K, (0.769 ± 0.005) × 10^−3^ s, (Figure 2b; Figure S9, Supporting Information). To further rationalize the fitting parameter Δ*E*, namely describing the nature of the thermally activated nonradiative channel, an energy diagram was built (Figure 2d). The partial diagram includes the Eu^3+^ and Tb^3+^ levels, the d‐U(600) excited singlet (S^H^) and triplet (T^H^) states^22^ and those from the tfac ligand (S_1,2_ and T_1_).^23^ The energy difference between the barycenters of the ^5^D_4_ excited state and the low‐lying triplet, the T_1_ triplet of tfac ligand, matches the Δ*E* estimated from Equation (1), suggesting that this level is the nonradiative thermal activated channel for the Tb^3+^ ions. As theoretical calculations demonstrated ligand‐to‐Ln^3+^ energy transfer rates are one order of magnitude larger than the values estimated for direct hybrid‐to‐Ln^3+^ energy transfer,^26^ and so the dominant intramolecular energy transfer pathway involving the Tb^3+^ levels is $S_{1}^{H}$ →$T_{1}^{H}$ → T_1_ → ^5^D_4_ → ^7^F_6–0_, for excitation in the d‐U(600) singlet excited states. Despite the resonance between T_1_ and $T_{1}^{H}$, this later level is not considered thermally coupled with the ^5^D_4_ level due to the quite effective $T_{1}^{H}$ → T_1_ energy transfer rates, both through the multipolar and exchange energy transfer mechanisms.^26^ For Eu^3+^ ions, the exchange mechanism is the dominant energy transfer mechanism and for excitation in the d‐U(600) singlet excited states the most efficient channel is $S_{1}^{H}$ → $T_{1}^{H}$ → T_1_ → (^5^D_1_, ^5^D_0_) → ^7^F_0–6_. The Δ*E* value experimentally accessed from the data best fit using Equation (1) is an average value for the barycenters energy separation taking into account the temperature dependence of the population distribution among T_1_‐related vibrational levels and high energy ^5^D_4_ Stark components.

In what follows, we show how luminescence thermometry is on the route of mobile‐based IoT. The abovementioned energy transfer mechanism involving the coupling between the T_1_ and ^5^D_4_ levels is exploited to demonstrate that the G/R ratio acquired through a smartphone and used to determine the absolute temperature is the first ever reported example of an intramolecular primary thermometer.

Accordingly to the well‐known relation between the luminescence intensity of a given transition and the lifetime of the upper transition level^27^ (Equation S4 in Supporting Information), the thermal dependence of the ^5^D_0_ → ^7^F_2_ (*I*_Eu_) and ^5^D_4_ → ^7^F_5_ (*I*_Tb_) transitions (Figure 2c) should be analogous to that found for τ(*T*) of the ^5^D_0_ and ^5^D_4_ states (Figure 2b). This is observed in the 280–317 K range. Moreover, it is well established in the literature that the sagest definition of a thermometric parameter should consider a ratio of intensities to compensate optical drifts and concentration fluctuations.^14^, ^15^ Because *I*_Eu_ is temperature independent, the ratiometric thermometric parameter Δ = *I*_Tb_/*I*_Eu_ has the same functional form of Equation (1), (Equations S5–S7 in Supporting Information). For further convenience, we consider a normalized thermometric parameter as

(2) $$\Delta_{N}\;\;\equiv \;\;\frac{\Delta_{0}}{\Delta }\;\;-\;\;1\;\;=\;\;\alpha \exp\left(-\frac{\Delta E}{k_{B}T}\right)$$

where *Δ*_0_ is the intensity ratio at the limit *T* → 0 K. We notice that the temperature dependences of Δ_N_ and τ_N_ ≡ τ/τ_0_ − 1 are described by the same α and Δ*E* parameters, meaning the temperature values can be predicted trough Equation (2) avoiding the conventional intensity‐to‐temperature laborious calibration. To circumvent the phenomenological character of the α parameter, Equation (2) may be rewritten expressing α as function of one known temperature, *T*_0_, to determine the corresponding Δ_N_ value at *T*_0_, denoted by Δ_N0_. The temperature is, then, calculated through

(3) $$\frac{1}{T}\;\;=\;\;\frac{1}{T_{0}}\;\;-\;\;\frac{k_{B}}{\Delta E}\;\;\times \;\;\ln\left(\frac{\Delta_{N}}{\Delta_{N0}}\right)$$

**Figure 3:**
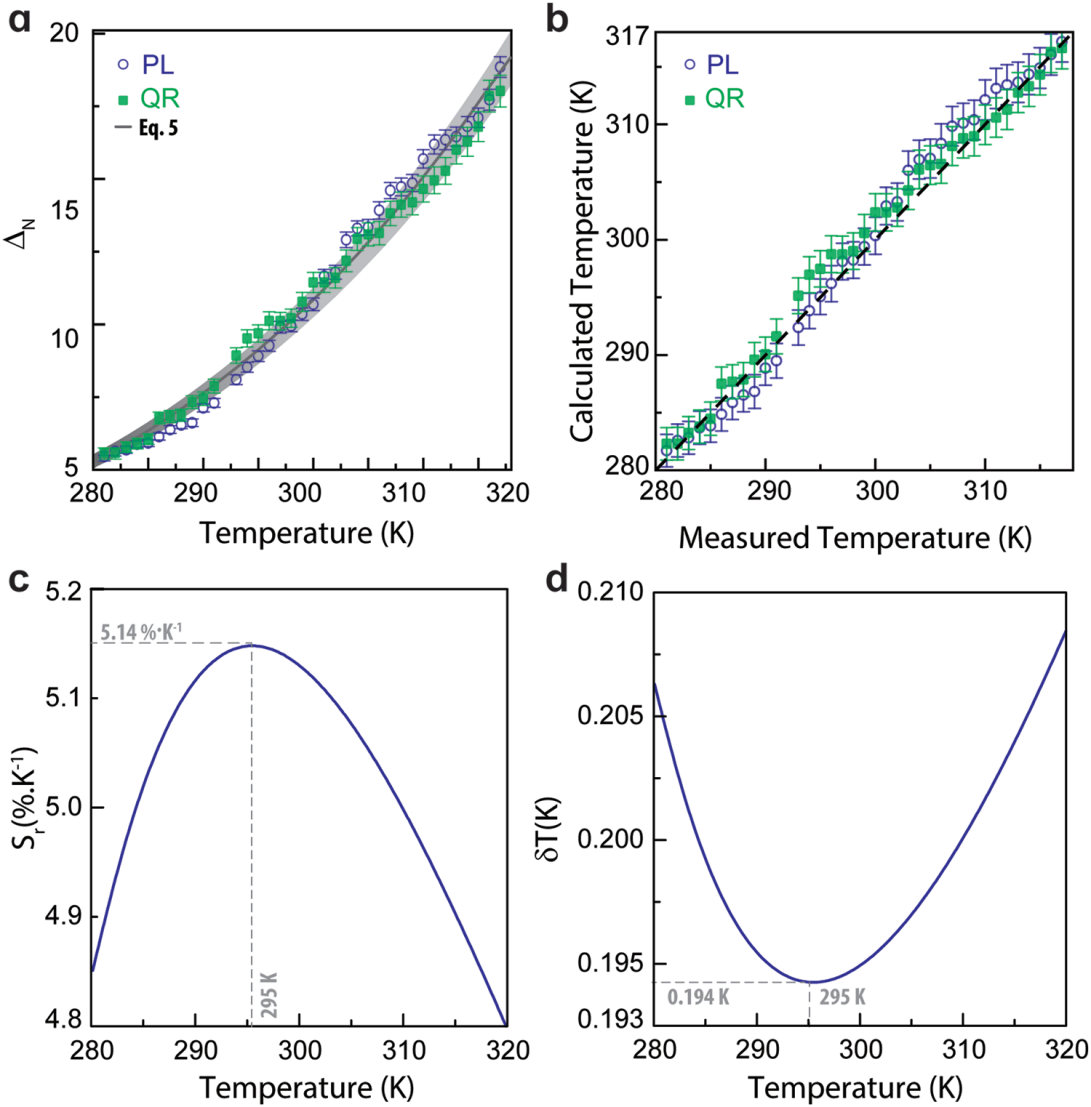
a) Calibration curve of the QR codes using the emission spectra (Δ_N_^PL^) and a photograph recorded by a smartphone (Δ_N_^QR^) in the 281–317 K range. The solid line is the predicted calibration curve of the primary intramolecular curve (Equation (3)). b) Comparison between the calculated (*y*) and measured (*x*) temperature values. The calculated temperatures use the emission spectra (PL) or the image of the QR code (QR). The line corresponds to *y* = *x*. The relative thermal sensitivity and temperature uncertainty in the same temperature range are presented in (c) and (d), respectively.

We want to emphasize that rewriting the thermometric parameter as Δ_N_ the system follows the same functional dependence previously reported by some of us for luminescent thermometers described by two thermally coupled emitting levels ruled by the Boltzmann law.^28^ To validate the use of Equation (3) to calculate the absolute temperature, we first use the well‐established analysis of the emission spectra in the 281–317 K range. The integrated emission areas, and the corresponding uncertainties (Equation S11, Supporting Information), were evaluated using the signal‐to‐noise ratio of the emission spectra, following the strategy detailed in the literature^14^ (Figure S10, Supporting Information). The ratio of integrated intensities calculated from the emission spectra is denoted by Δ^PL^ and its value in the limit *T* → 0 K is determined using the emission spectra recorded at 12 K, yielding to Δ_0_^PL^ = 2.40 ± 0.05 (Figure S7, Supporting Information). Next the normalized thermometric parameter (Δ_N_^PL^) is calculated and represented with respect to the measured temperature (**Figure** **3**a) with Δ_N0_ calculated through the emission spectrum recorded at *T*_0_ = 281 K. The Δ_N_ curve predicted by Equation (3) is represented for comparison in the same figure. We observe an excellent agreement between the experimental values and those predicted, validating Equation (3) as the equation of state for this thermometer. The repeatability of the QR code was tested in three consecutive temperature cycles and the maximum repeatability is 97% at 283 K (Figure S11, Supporting Information).

Envisaging the temperature sensing using the processing of a smartphone's photographic record of the code we correlate the changes in the emission spectra with the changes in the RGB color coordinates of the photographs. The thermometric parameter (Equation (4)) is accessed by the ratio between the green (*G*) and red (*R*) channels presented in Figure 2a

(4) $$\Delta^{\mathrm{QR}}\;\;=\;\;\frac{G}{R}$$

and the corresponding uncertainty (Equation (5)) is

(5) $$\delta \;\Delta^{\mathrm{QR}}\;\;=\;\;\Delta^{\mathrm{QR}}\;\sqrt{{\left(\frac{\delta R}{R}\right)}^{2}\;\;+\;\;{\left(\frac{\delta G}{G}\right)}^{2}}$$

The thermometer performance can be quantified using the relative thermal sensitivity and temperature uncertainty (details of calculus in the Supporting Information). The maximum relative sensitivity (*S*_m_) and the minimum temperature uncertainty of the QR code are 5.14%·K^−1^ and 0.194 K, respectively, both at 293 K, among the best values reported for luminescent thermometers (secondary and primary) based on Ln^3+^ ions, including examples of upconverting nanoparticles, molecular systems, metal‐organic frameworks, and near infrared emitters (for comparison of the *S*_m_ values see Table 1 of ref. 15, Figure 4 of ref. 29, Table 10.3 of ref. 30, and Figure S12a in the Supporting Information).

Attending to the thermal dependence of the *S*_r_ values with the parameters that characterize the intramolecular luminescent primary thermometer, we calculate the curves resulting from values of Δ*E* between 500 and 5500 cm^−1^ (the energetic separation value between the ^5^D_4_ and the ^5^D_3_ level, Figure S12b in the Supporting Information). The maximum energy value was chosen to simulate the situation of the triplet state responsible to the ^5^D_4_ depopulation starts to depopulate the next level in the Tb^3+^ energy diagram. It points out to the possibility of choosing the temperature range of operation of these thermometers playing with Δ*E* value: the lower the Δ*E*, the lower the *T*_m_, and the higher *S*_m_ values. For thermometers operating in the temperature range of interest for IoT applications (typ. 270–330 K range, shadowed area in Figure S12b in the Supporting Information), Δ*E* should lie between 3500 and 4000 cm^−1^. The thermometer reported here is characterized by Δ*E* = 3534 ± 218 cm^−1^ exactly within the optimum range that produces the higher *S*_m_ values in the temperature range of interest.

After establishing the concept of QR codes as smart labels for sensing, a practical example for temperature decoding was performed through the development of a free access and user‐friendly mobile application for smartphones (mobile app, further details in Figure S13 in the Supporting Information). This app enables the simultaneous QR code decoding and temperature reading through the pixel intensity map of one photograph of the luminescent QR code captured by the camera of the smartphone. One luminescent QR code photo is taken and the application simultaneously decodes the QR code information and indicates the temperature at which the photo was captured. The app is free and available for download at https://tinyurl.com/qr‐luminescent.

## 3 Conclusions

Luminescent QR codes were fabricated with Eu^3+^/Tb^3^‐doped organic–inorganic hybrid materials. Photographs taken with the CCD camera of a mobile phone enable the temperature readout with a maximum relative sensitivity and a minimum uncertainty of 5.14% K^−1^ and 0.194 K, respectively, both at 293 K. These figures of merit result from the thermal coupling between the ^5^D_4_ Tb^3+^‐excited level and the low‐lying triplet states of the organic ligands and are among the best ones known for luminescent thermometers. The ratio between the intensity of the green and red pixels of the photos are the basis for the temperature sensing through a unique intramolecular primary thermometer opening the possibility for the implementation of QR codes in mobile IoT without the need of any technological adaptation of current smartphones.

This is the first example in which smartphones are used as an effective alternative to portable spectrometers to calculate the temperature using the induced color temperature change. The methodology constitutes an innovation in the area, assigning technological value to the QR codes and leveraging the area of IoT devices toward smart labels using a smartphone in its original configuration, as there is no need to adapt neither the tag decoding nor the CCD detector for temperature sensing, in which e‐health is a target application. Additional features are envisaged to the luminescent QR codes (e.g., traceability, data storage, and security alerts) through dedicated applications, that establish connections and information exchanging between the QR code reader and the cloud, in which the encrypting connection may appear as an optional tool. A dedicated mobile app was build enabling the simultaneous QR code information access and the temperature monitoring, demonstrating the unequivocal applicability of the developed concept behind the QR codes as smart labels in IoT.

## 4 Experimental Section

*Materials*: The binuclear Eu_0.25_Tb_0.75_(tfac)_3_ ⋅ H_2_O complex (tfac = 1,1,1‐trifluoro‐2,4‐pentanedione, Sigma‐Aldrich), was synthesized as previously reported,^9^ (further details in Figure S1 in the Supporting Information). Elemental analyses for C and H were performed with a TruSpec 630‐200‐200 CNHS Analyzer. The doping concentrations of Eu^3+^ and Tb^3+^ were determined by ICP‐OES (inductively coupled plasma‐optical emission spectroscopy) on a Horiba Jobin Yvon model ACTIVA M. The analytical results were: calcd. (wt%): C 28.38, H 2.21, Eu 5.99, and Tb 18.80; found (wt%): C 27.97, H 2.24, Eu 5.97, and Tb 18.10. To process the complex as films it was incorporated into a diureasil organic–inorganic hybrid host, so‐called d‐U(600), which was formed by polyether chains (with average molecular weight of 600 g ⋅ mol^−1^) covalently linked to a siliceous inorganic skeleton by urea bridges, as previously reported^31^ (further details in Figure S1 in the Supporting Information). The resulting material, hereafter termed as dU6EuTb, was characterized by X‐ray diffraction (XRD) and Fourier transform infrared (FT‐IR) spectroscopy, as detailed in Figure S2 in the Supporting information.

*QR Codes Processing*: Luminescent QR codes version 1 (21 × 21 modules^2^) with error correction level L (7% of codewords can be restored by a Reed‐Solomon error algorithm) and dimensions 5 × 5 cm^2^ with the different messages, “SMART LABELLING,” “UNIVERSITY OF AVEIRO,” and “INST. DE TELECOMUNICACOES,” were implemented. Aiming at preparing a luminescent layer, QR codes produced on a 5.0 × 10^−4^ m thickness acetate substrate layer were laser cut (the acetate on the inactive modules region was removed). These QR codes were vertically immersed in a solution of the dU6EuTb (3.39 wt%) at a velocity of 1.4 × 10^−3^ m s^−1^ using a homemade dip‐coating system. After the deposition, the substrates with the luminescent QR codes were transferred to an oven at 45 °C for 48 h. The QR codes were transparent under day light, enabling color‐based multiplexing. This strategy to multiplex distinct colored QR codes consists in overlapping a conventional black/white QR code by the luminescent QR code. Under daylight the acetate‐based luminescent code was transparent, and the base code was readily accessed, whereas under UV illumination the acetate‐based QR code becomes luminescent (Figure 1a,b), enabling the color‐multiplexing of the overlapped codes, as recently proposed.^9^

*Optical Characterization—Image Acquisition*: The photographs of the luminescent QR codes under UV illumination were taken with a smartphone camera with resolution of 2238 × 3986 pixel^2^, aperture of *f*/2 and a sensor dimension of 1/4.2″.

*Optical Characterization—Emission Spectra and Temperature Calibration*: The temperature‐dependent emission spectra of the QR codes were measured using a portable spectrometer (OceanOptics Maya 2000 Pro) coupled with an optical fiber, under a UV lamp excitation (254 nm). The QR code response to temperature was calibrated using either the emission spectra or the smartphone photographic records of the codes. For calibration, a homemade Peltier‐plate based temperature controller and a K‐type thermocouple were used. The temperature was set in the temperature controller and the QR code was let to thermalize for 5 min to ensure a constant temperature value reading in the thermocouple. An emission spectrum (integration time of 1.0 s, recorded at a central location of the code) and a photograph of the whole code were collected using the portable spectrometer and the smartphone's camera, respectively. The procedure was repeated in the 283–323 K range (step of 1 K). To independently monitor the temperature attesting its uniformity within the QR code surface, a thermographic camera FLIR DG001U‐E (sensitivity of 0.1 K, accuracy of ±2 K, according to the manufacturer) was used. The IR camera temperature profiles result from an averaging of four thermal images acquired in distinct regions of the QR code (Figure S3, Supporting Information). To estimate the dU6EuTb layer emissivity (ε), the procedure described elsewhere was adopted,^9^ resulting ε = 0.85. The emission color coordinates were calculated from the emission spectra using the 1931 Commission Internationale de Éclairage (CIE) methodology defined for the 2nd standard, whereas the color coordinates from the photographs were determined using the RGB model (Supporting Information for details).

*Photoluminescence*: The emission and the excitation spectra were recorded using a modular double grating excitation spectrofluorimeter with an emission monochromator (Fluorolog‐3 2‐Triax, Horiba Scientific) coupled to a photomultiplier (R928 Hamamatsu), using the front face acquisition mode. The excitation source was a 450 W xenon arc lamp. The excitation spectra were weighed for the spectral distribution of the lamp intensity using a photodiode reference detector. The absolute emission quantum yields were measured at room temperature using a system (Quantaurus‐QY Plus C13534, Hamamatsu) with a 150 W xenon lamp coupled to a monochromator for wavelength discrimination, an integrating sphere as the sample chamber, and a multichannel analyzer for signal detection. The method was accurate to within 10%.

*RGB Color Model*: The RGB color model is an additive model that creates color through the mix of three primaries colors Red, Green, and Blue. Based on this every image can be decomposed into that three channels, one corresponding to each primary being possible afterward to determine the mean value for each channels (*µ*_R_,_G,B_) using an histogram fitted with a Gaussian function and their associated error defined by the standard error of the mean, Equation (6)

(6) $$\Delta \mu_{R,G,B}=\frac{\sigma_{R,G,B}}{\sqrt{n}}$$

where *n* is the sample number of points and σ_R,G,B_ is the standard deviation of each fit.

The R, G, and B values were normalized using Equation (7)

(7) $$r\;\;=\;\;\frac{R}{R\;\;+\;\;G\;\;+\;\;B};\;g\;\;=\;\;\frac{G}{R\;\;+\;\;G\;\;+\;\;B};\;b\;\;=\;\;\frac{B}{R\;\;+\;\;G\;\;+\;\;B}$$

## Conflict of Interest

The authors declare no conflict of interest.

## Supporting information

Supplementary
