# Supplementary material for: Luminescence Thermometry on the Route of the Mobile‐Based Internet of Things (IoT): How Smart QR Codes Make It Real
Source: Adv Sci (Weinh). 2019 Aug 9;6(19):1900950. doi: 10.1002/advs.201900950 (PMC6774024; doi:10.1002/advs.201900950)
Supplement: Supplementary file 1 — Supplementary [file ADVS-6-1900950-s001.pdf]

Copyright WILEY-VCH Verlag GmbH & Co. KGaA, 69469 Weinheim, Germany, 2019.

ADVANCED SCIENCE
Open Access

Supporting Information

for *Adv. Sci.,* DOI: 10.1002/advs.201900950

Luminescence Thermometry on the Route of the Mobile-Based Internet of Things (IoT): How Smart QR Codes Make It Real

João F. C. B. Ramalho, Sandra F. H. Correia, Lianshe Fu, Lara L. F. António, Carlos D. S. Brites, Paulo S. André, Rute A. S. Ferreira,* and Luís D. Carlos*

Luminescence thermometry on the route of the mobile-based Internet of Things (IoT): How smart QR codes make it real

João F.C.B. Ramalho[1,2], Sandra F.H. Correia[1], Lianshe Fu[1], Lara C.F. António[1,2], Carlos D.S. Brites[1], Paulo S. André[2,3*], Rute A.S. Ferreira[1*] and Luís D. Carlos[1]

[1] Department of Physics and CICECO - Aveiro Institute of Materials, University of Aveiro, 3810-193 Aveiro, Portugal
[2] Department of Electronics, Telecommunications and Informatics, Instituto de Telecomunicações, University of Aveiro, 3810-193 Aveiro, Portugal
[3] Department of Electric and Computer Engineering and Instituto de Telecomunicações, Instituto Superior Técnico, Universidade de Lisboa, 1049-001 Lisbon, Portugal

S1. Materials 2

S1.1 Synthesis of $Eu_{0.25}Tb_{0.75}(tfac)_3{\cdot}H_2O$ complex. 2

S1.2 Synthesis of di-ureasil, dU(600), doped with the $Eu_{0.25}Tb_{0.75}(tfac)_3{\cdot}H_2O$ complex. 2

S1.3 Structural characterization 3

S2. Temperature monitoring with IR thermal camera 4

S3. Colour identification 5

S4. Photoluminescence 8

S4.1 Excitation spectra 8

S4.2 Emission spectra 9

S4.3 Emission decay curves 11

S5. Temperature Calibration 13

S5.1. Thermometric parameter 13

S5.2. Relative thermal sensitivity and temperature uncertainty 14

S5.3 Repeatability 16

S5.4 Primary Thermometers 16

S5.5 QR code mobile App 18

Supplementary References 18

* Corresponding authors.

Email address: Ferreira, R.A.S. (rferreira@ua.pt); P.S. André (paulo.andre@av.it.pt).

S1. Materials

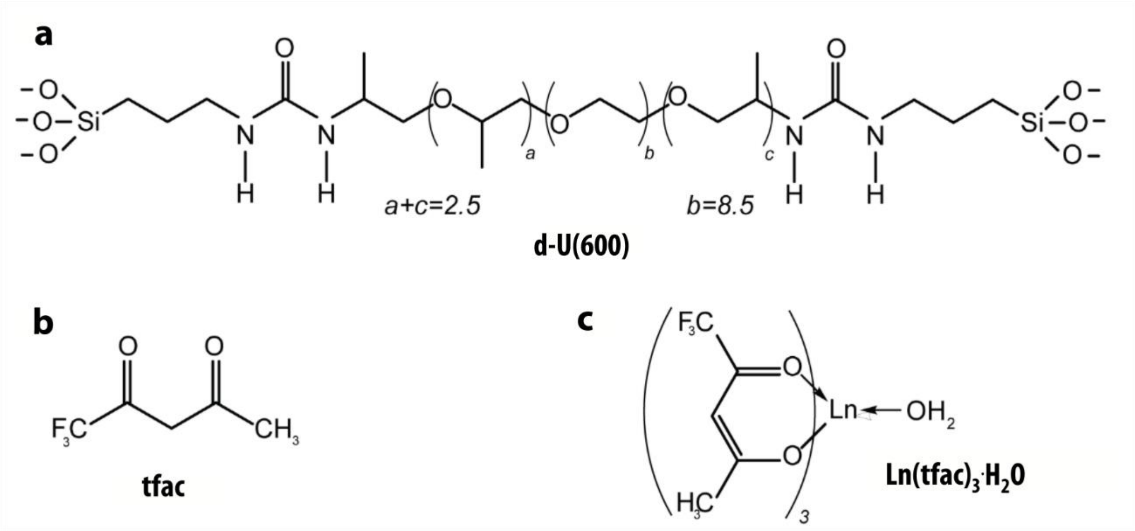

Figure S1. Molecular structures of **(a)** d-U(600), **(b)** tfac ligand and of the mononuclear **(c)** Ln(tfac)$_3$·H_2O (Ln=Eu or Tb) complex.

S1.1 Synthesis of $Eu_{0.25}Tb_{0.75}(tfac)_3\cdot H_2O$ complex.

The $Eu_{0.25}Tb_{0.75}(tfac)_3\cdot H_2O$ was synthesized according to the literature.[1] Typically, 0.74 mL (6.0 mmol) of 1,1,1-trifluoroacetylacetone (tfac, Sigma–Aldrich) was transferred to 10 mL of distilled water, followed by addition of 6.0 mL (6.0 mmol) of 1.0 mol/L $NH_3\cdot H_2O$. The resulting mixture was stirred at room temperature until a homogenous clear solution was obtained. Then 183.2 mg (0.5 mmol) of $EuCl_3\cdot 6H_2O$ and 560.1 mg (1.5 mmol) of $TbCl_3\cdot 6H_2O$ were added with the molar ratio of Eu and Tb to tfac is 1:3 and the precipitate appeared. The mixture was further stirred at 50 ºC and then placed at ambient condition overnight. The resultant precipitate was filtered off, washed with water and dried.

S1.2 Synthesis of di-ureasil, dU(600), doped with the $Eu_{0.25}Tb_{0.75}(tfac)_3\cdot H_2O$ complex.

The organic-inorganic hybrid precursor, d-UPTES(600), was prepared according to the literature.[2] In order to get the optimal doping concentration for the QR code fabrication, two doping contents corresponding to the final concentrations in the gels of 0.87 wt% (dU6TbEu-1) and 3.39 wt% (dU6TbEu), were adopted. For synthesis of sol doped with higher concentration of $Eu_{0.25}Tb_{0.75}(tfac)_3\cdot H_2O$, typically, 6.0 g (5.484 mmol) of d-UPTES(600) was mixed with 8 mL of EtOH under stirring. Then 168.0 mg of $Eu_{0.25}Tb_{0.75}(tfac)_3\cdot H_2O$ was added, and the mixture was treated under ultrasonic condition until a clear solution was obtained. Next 0.592 mL of HCl acidified water (pH=2) was added under stirring to catalyse the hydrolysis and condensation

reactions. The molar ratio of d-UPTES(600):H_2O is 1:6. The resulting sol (dU6EuTb) was stirred at room temperature for further 2 hours and then it was deposited by dip-coating, as detailed below. The resulting materials were characterised by X-ray diffraction (XRD) and Fourier transform infrared (FT-IR) spectroscopy, as detailed in the following section.

S1.3 Structural characterization

The powder X-ray diffraction (XRD) patterns were recorded in the 2θ range spanning from 3.5 to 70.0° by using a Panalytical Empyrean Diffractometer under exposure of CuK_{α} radiation (λ= 1.54 Å) at room temperature, Figure S2A. The $Eu_{0.25}Tb_{0.75}(tfac)_3{\cdot}H_2O$ diffraction pattern reveals a crystalline structure, whose determination will be further studied. We notice that the tentative chemical structure consisting of one water molecule is in accordance with previous results[3, 4] and with elemental analysis (experimental section on the manuscript). However, the presence of more than water molecule cannot be excluded.

The XRD pattern for dU6EuTb-1 (lower doping concentration of $Eu_{0.25}Tb_{0.75}(tfac)_3{\cdot}H_2O$ is dominated by a broad band centered at around 21°, that is characteristic of amorphous structure of di-ureasils.[5] The peaks related to the complex are not evident in the pattern of dU6EuTb-1, due to the low relative concentration (0.87 wt%) of the complex in dU6TbEu-1 in comparison with dU6TbEu (3.39 wt%).

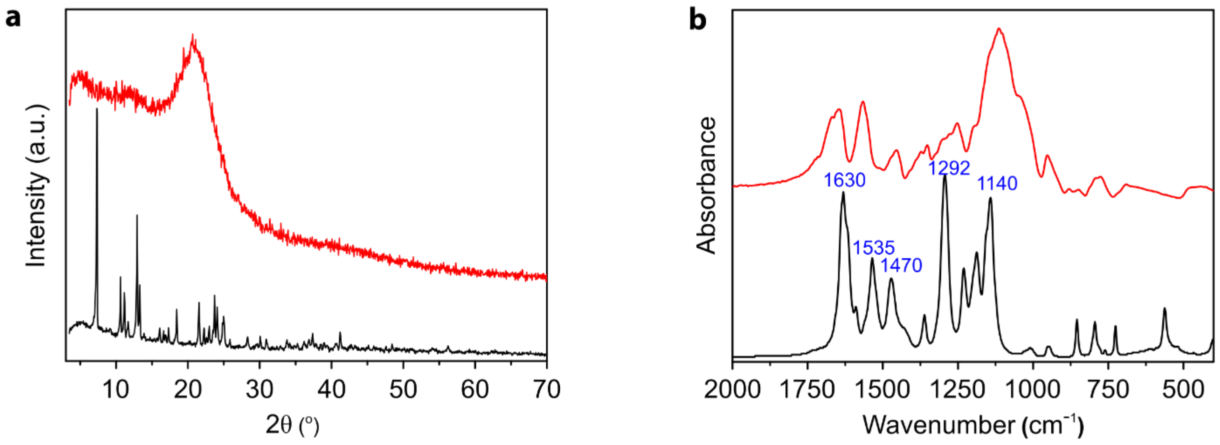

Figure S2. (a) XRD patterns and **(b)** FT-IR spectra of the (black line) $Eu_{0.25}Tb_{0.75}(tfac)_3{\cdot}H_2O$ complex and of (red line) dU6EuTb-1.

The Fourier transform infrared (FT-IR) spectra were obtained using a MATTSON 7000 FT-IR Spectrometer system to scan the sample absorbance intensity from 4000–400 cm^{-1} with 64 scans

and 2 cm^{-1} resolution (Figure S2B). The FT-IR spectrum of $Eu_{0.25}Tb_{0.75}(tfac)_3{\cdot}H_2O$ shows C=O and C=C stretching vibrations at 1630 and 1535 cm^{-1}, respectively, being characteristic absorptions of Ln^{3+}-based β-diketonates due to the extended π-conjugation structure.[3] The peak at 1470 cm^{-1} is from CH bending vibration combining with C=O stretching vibrations. The two strong absorption bands at 1292 and 1140 cm^{-1} in the complex spectrum are assigned to asymmetric and symmetric stretching vibrations of CF_3 groups, respectively.[6] The vibration bands of Ln–O are not observed in the recorded region. Moreover, because of the low concentration (0.87 wt%) of the complex in dU6EuTb-1 and/or due to the overlap of the weaker absorption band from di-ureasil, the absorption bands from the complex are not detected in dU6EuTb-1 spectra. Thus, there are no evidences that the d-U(600) coordinates to the Ln^{3+} ions.

S2. Temperature monitoring with IR thermal camera

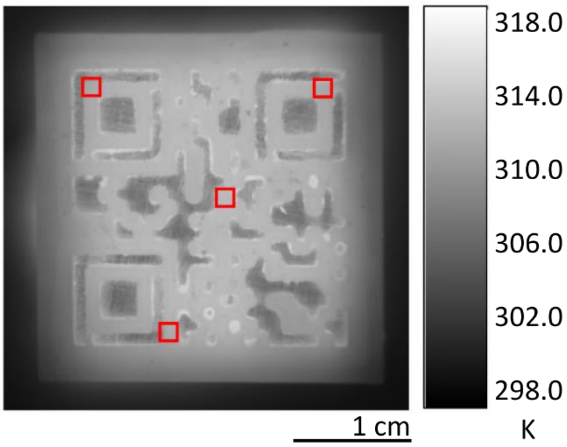

Figure S3. Photograph of a luminescent QR code coated with dU6EuTb recorded by the thermal camera FLIR DG001U-E. The regions marked in red are the four zones where the temperature was measured to verify the uniformity within the QR code surface.

S3. Colour identification

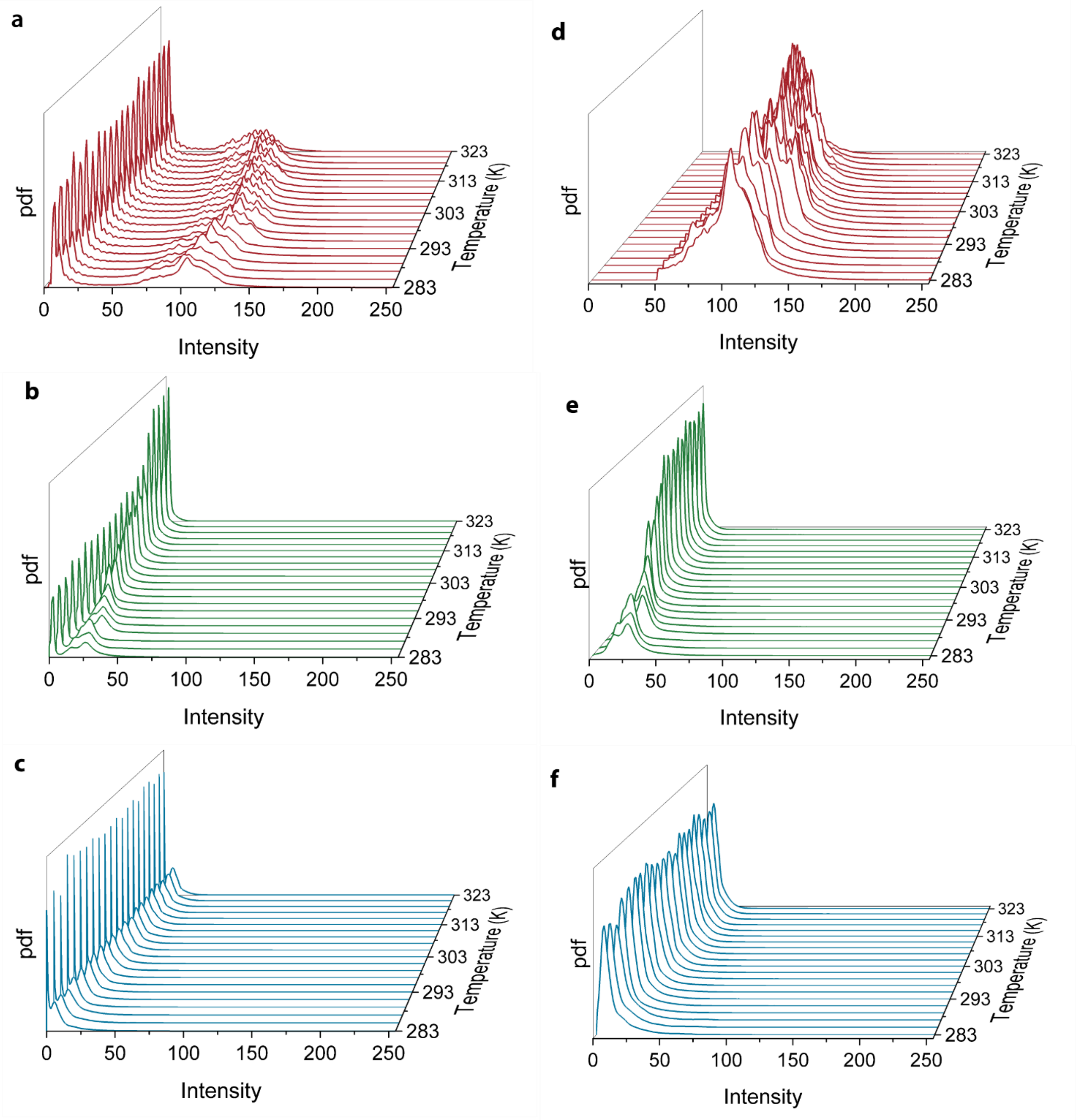

Figure S4. (a) Red, **(b)** green and **(c)** blue components histograms variation with temperature in the 283 to 323 K range, calculated from photographic records of the luminescent QR codes. **(d)**, **(e)** and **(f)** selected histograms without taking into account the black colour pixels.

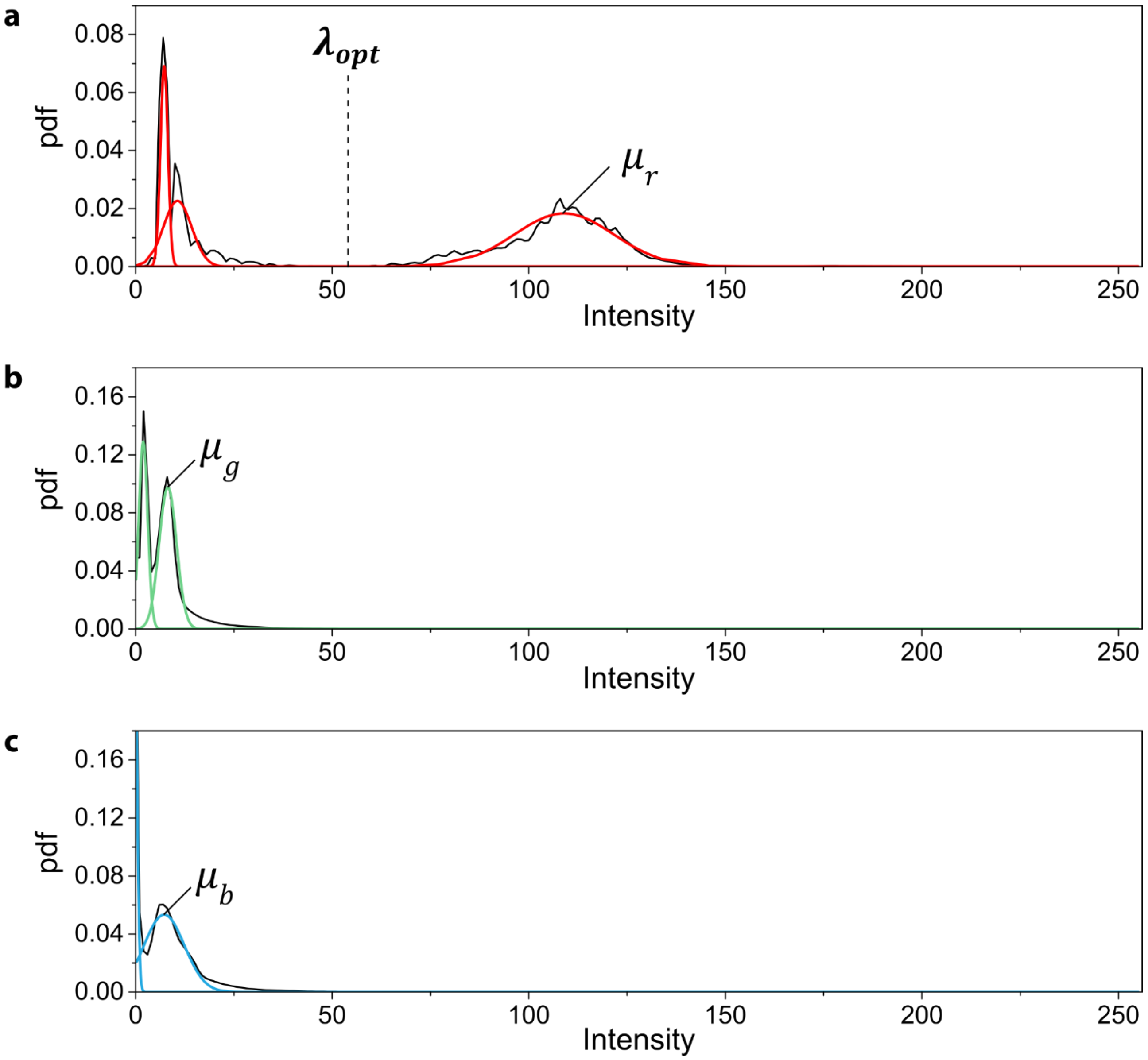

Figure S5. (a) Red, **(b)** green and **(c)** blue components histograms (black lines) for the luminescent QR code photographic record retrieved at 303 K modulated (*r*>0.99) by Gaussian functions (red, green and blue lines, respectively).

The peaks near the zero-intensity value in all histograms corresponds to the black modules, represented by coordinates (0,0,0), while the red active modules are responsible by the maximum intensity value in the R component. We note that the emission colour from the luminescent material is not pure (R or G) thus it is related to the peak in R histogram at intensity 110 but also to small contributions on the remaining primaries G (~38 decreasing with temperature) and B (~6), this is explained with the mismatch between the material emission spectra and the RGB space primaries wavelength ranges.

Considering only the R component histogram, composed of two peaks fitted with gaussian *pdf* centred at A and B, with variance σ_A^2 and σ_B^2 , the error probability when separating the colours, and considering that each colour has a probability of p_A and p_B, is given by:[2]

$$p_{error}(\lambda) = \frac{p_B}{\sqrt{2\pi\sigma_B^2}} \int_{-\infty}^{\lambda} \exp\left(-\frac{(x-B)^2}{2\sigma_B^2}\right) dx + \frac{p_A}{\sqrt{2\pi\sigma_A^2}} \int_{\lambda}^{\infty} \exp\left(\frac{(x-A)^2}{2\sigma_A^2}\right) dx \qquad \text{(S1)}$$

*w*here λ is the decision level. Minimizing the error probability allow to determine the ideal decision level, λ_{opt}, level, and is given by:

$$\begin{cases} \lambda_{opt} = \frac{A+B}{2}, \sigma_A = \sigma_B \wedge p_A = p_B \\ \lambda_{opt} = \frac{-\left(A\sigma_B^2 - B\sigma_A^2\right) \pm 2\sqrt{(A\sigma_B^2 - B\sigma_A^2)^2 - (\sigma_A^2 - \sigma_B^2)\left(B^2\sigma_A^2 - A^2\sigma_B^2 - 2\sigma_A^2\sigma_B^2 \ln\left(\frac{p_B\sigma_A}{p_A\sigma_B}\right)\right)}}{(\sigma_A^2 - \sigma_B^2)}, \sigma_1 \neq \sigma_2 \wedge p_A \neq p_B \end{cases} \qquad \text{(S2)}$$

In the case both colour have the same probability values ($p_A = p_B$) as well as the same variance ($\sigma_A^2 = \sigma_B^2 = \sigma^2$) the error probability is given by:

$$p_{error} = \frac{1}{2} erfc\left(\frac{B-A}{2\sqrt{2}\sigma^2}\right) \qquad \text{(S3)}$$

where *erfc (.)* refers to the complementary error function

S4. Photoluminescence

S4.1 Excitation spectra

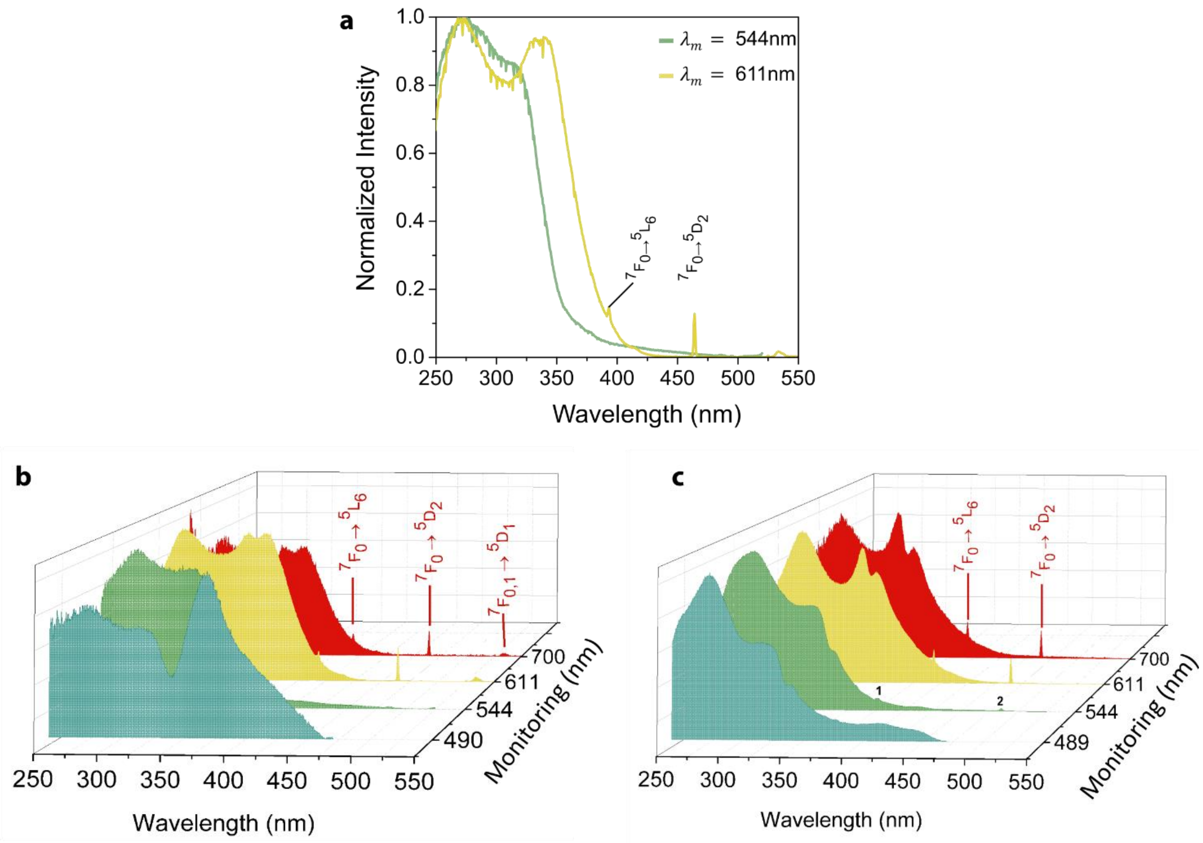

Figure S6. (a) Comparison between the room temperature excitation spectra of the dU6EuTb monitored within the Tb^{3+} (544 nm) and the Eu^{3+} (611 nm) related emission. Excitation spectra monitoring different wavelengths acquired at **(b)** 298 K and at **(c)** 12 K.

The excitation spectra of the dU6EuTb (Figure S6) spectra resemble those of the single-based complexes[7] being observed a larger full-width-at-half maximum (fwhm) for the excitation spectra monitored within the Eu^{3+} emission in the bi-nuclear $Eu_{0.25}Tb_{0.75}(tfac)_3{\cdot}H_2O$ and mono-nuclear $Eu(tfac)_3{\cdot}H_2O$ complexes[7] compared with those monitored within the Tb^{3+} lines (both in $Eu_{0.25}Tb_{0.75}(tfac)_3{\cdot}H_2O$ and mono-nuclear $Tb(tfac)_3{\cdot}H_2O$ complexes[7]). Although the clarification of this aspect lies beyond the scope of this work, this larger fwhm suggests the presence of another component (around 370 nm) dedicated to the selective excitation of intra-$4f^6$ levels, tentatively ascribed to a ligand-to-metal-charge transfer (LMCT) band. As the energy of LMCT states is dependent on the Ln^{3+} ion (Tb^{3+}-based LMCT bands are blue-shift when compared with those involving Eu^{3+}, for the same ligands)[8] the non-observation of such component, while monitoring the intra-$4f^8$ levels is a supporting argument for the LMCT band assignment.

S4.2 Emission spectra

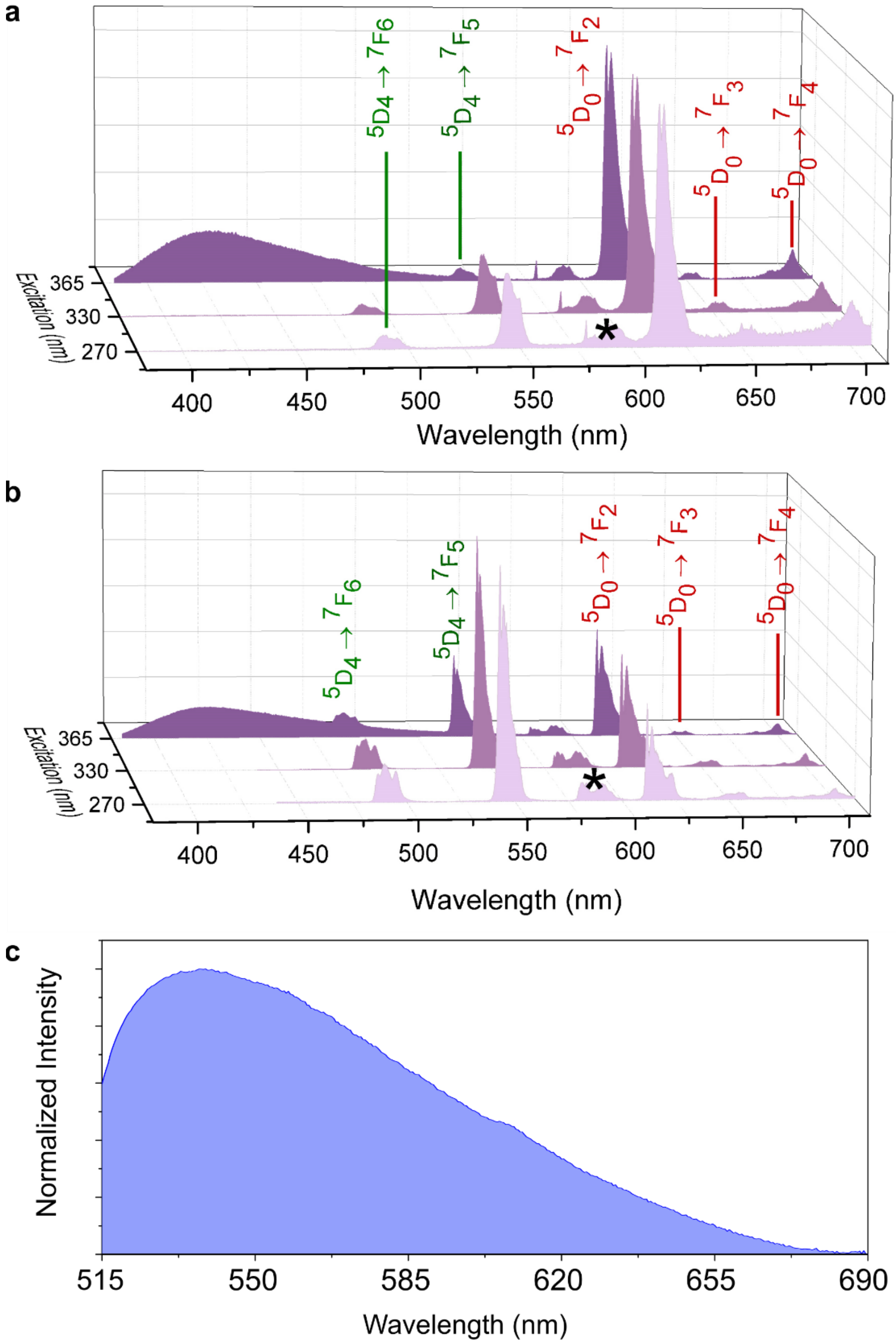

Figure S7. Emission spectra of the dU6EuTb at different excitation wavelengths acquired at **(a)** 298 K and at **(b)** 12 K. The asterisks denote the overlap between the $^5D_4 \rightarrow {}^7F_4$ (Tb^{3+}) and $^5D_0 \rightarrow {}^7F_{0,1}$ (Eu^{3+}) transitions. **(c)** Emission spectrum of the dU6EuTb excited under intra-$4f^8$ excitation wavelength (488 nm) acquired at 298 K.

Figure S7 shows the emission spectra obtained at 298 K and 12 K for dU6EuTb, excited at 270 nm, 330 nm and 365 nm. The narrow lines (fwhm <10 nm) are ascribed to the Tb^{3+} ($^5D_4 \rightarrow {}^7F_{3-6}$) and Eu^{3+} ($^5D_0 \rightarrow {}^7F_{0-4}$). The energy and fwhm of these transitions do not vary as a function of the excitation wavelength, suggesting a unique local environment for the Tb^{3+} and Eu^{3+} ions. For higher excitation wavelengths (in the case 365 nm), it should be noted the broad band (fwhm ~ 90 nm) between 380 and 580 nm, associated with the intrinsic emission of the hybrid[9] ascribed to the contribution of donor–acceptor pair recombinations occurring in the oxygen-related defects in the siliceous skeleton and proton transfer within the urea group.[10] The energy low-lying singlet and triplet states associated with the siliceous-based domains (S_1^{Si} and T_1^{Si}) and NH groups (S_1^{NH} and T_1^{NH}) indicated in the energy diagram of the manuscript were previously determined.[9-11] The phosphorescence spectra of the $Gd(tfac)_3H_2O$[12] is formed of a broad band in the blue region overlapping that of the intrinsic di-ureasil and, therefore, its contribution to the broad band in Figure S7 cannot be neglected.

S4.3 Emission decay curves

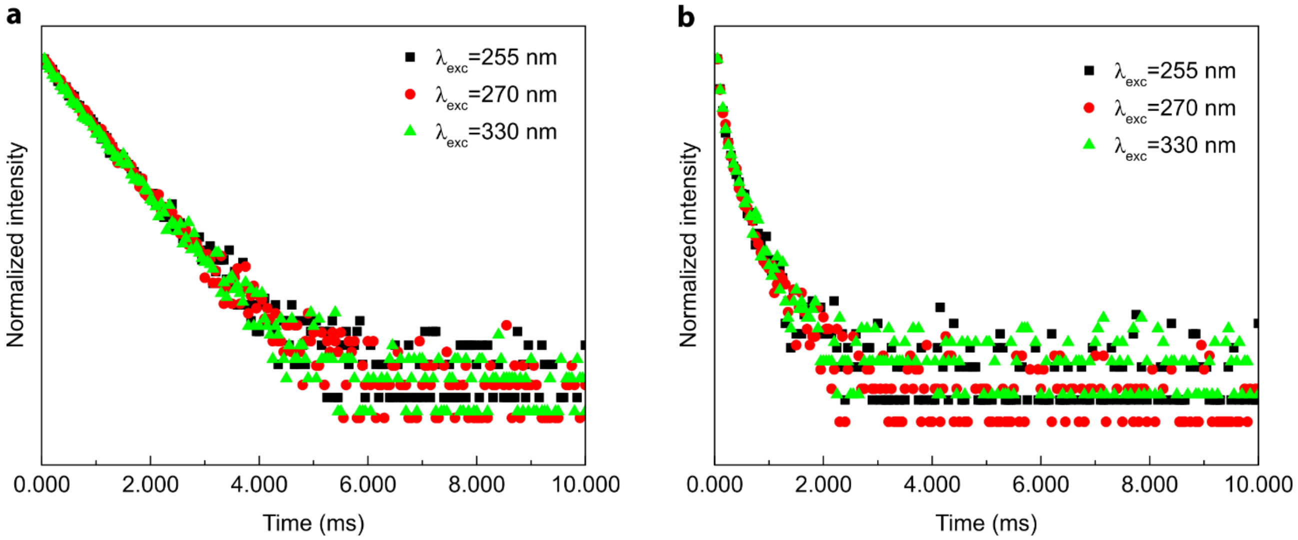

Figure S8. Emission decay curves (300 K) of the dU6EuTb excited at 255 nm, 270 nm and 330 nm and monitored at **(a)** 611 nm ($^5D_0 \rightarrow {}^7F_2$, Eu^{3+}) and at **(b)** 544 nm ($^5D_4 \rightarrow {}^7F_5$, Tb^{3+}).

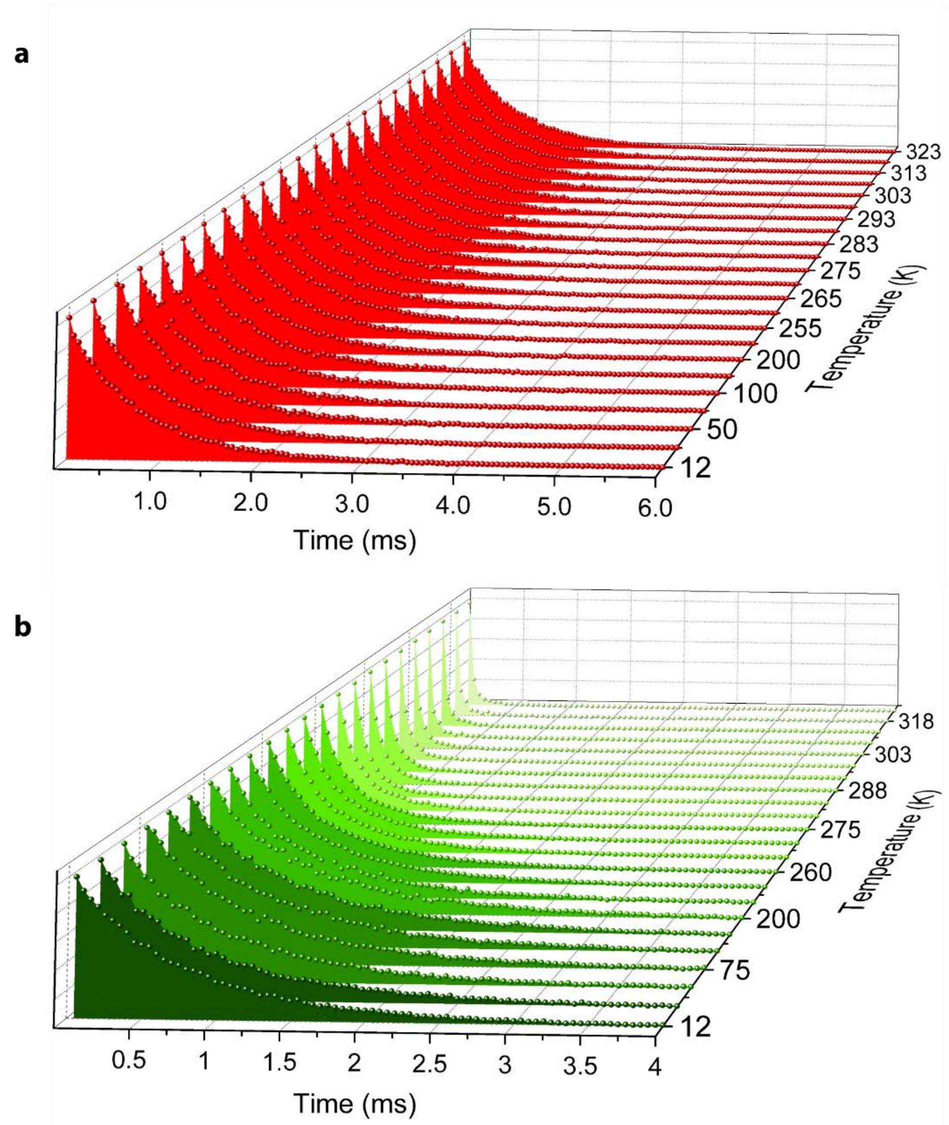

Figure S9. Thermal dependence of the emission decay curves of the dU6EuTb excited at 330 nm and monitored at **(a)** 611 nm ($^5D_0 \rightarrow {}^7F_2$, Eu^{3+}) and at **(b)** 544 nm ($^5D_4 \rightarrow {}^7F_5$, Tb^{3+}).

All the emission curves in Figures in S3.3 reveal a single exponential behavior, in good agreement with the selective detection of the emission decay from the $^5D_0$ (Eu^{3+}) and the $^5D_4$ (Tb^{3+}) and with the presence of a single average local environment for each ion.

S5. Temperature Calibration

S5.1. Thermometric parameter

The $\tau(T)$ dependence (Eq. 3 in the manuscript) is rationalized on the basis of the classical Mott-Seitz model.[13] When the integrated intensity $I(T)$ of a transition from level |1> to |0>, is proportional to the lifetime of |1> $\tau(T)$ that is modeled by Eq. 3 (in the manuscript), replacing $\tau(T)$ by $I(T)$:

$$I(T) = \frac{I_0}{1 + \alpha \exp\left(-\frac{\Delta E}{k_B T}\right)} \tag{S4}$$

where I_0 is the intensity at $T \rightarrow 0$ K.

Comparing the temperature dependence of $\tau(T)$ and of $I(T)$ in the 280–320 K temperature range, we notice that the thermal dependence of the $^5D_0 \rightarrow {}^7F_2$ (I_{Eu}) and of the $^5D_4 \rightarrow {}^7F_5$ (I_{Tb}) transitions (Figure 2c of the manuscript) is analogous to that found for the $\tau(T)$ of the $^5D_0$ and $^5D_4$ states (Figure 2b), validating the use of Eq. S5. Defining a ratiometric thermometric parameter by $\Delta = I_{Tb}/I_{Eu}$ we keep the same functional form of Eqs. 3 (manuscript) and S5. In this case the thermal dependence of Δ is:

$$\Delta = \frac{\Delta_0}{1 + \alpha \exp\left(-\frac{\Delta E}{k_B T}\right)} \tag{S5}$$

where Δ_0 is the limit of the intensity ratio at the limit $T \rightarrow 0$ K. For further convenience, we consider a normalized thermometric parameter as:

$$\Delta_N \equiv \frac{\Delta_0}{\Delta} - 1 = \alpha \exp\left(-\frac{\Delta E}{k_B T}\right). \tag{S6}$$

The temperature is calculated through:

$$\frac{1}{T} = \frac{1}{T_0} - \frac{k_B}{\Delta E} \times \ln\left(\frac{\Delta_N}{\Delta_{N0}}\right) \tag{S7}$$

and the temperature uncertainty is:

$$\delta T = T^2 \sqrt{\left(\frac{\delta T_0}{T_0^2}\right)^2 + \left(\frac{\delta \Delta E}{k_B} \ln\left(\frac{\Delta_N}{\Delta_{N0}}\right)\right)^2 + \left(\frac{k_B}{\Delta E}\right)^2 \left[\left(\frac{\delta \Delta_N}{\Delta_N^2}\right)^2 + \left(\frac{\delta \Delta_{N0}}{\Delta_{N0}^2}\right)^2\right]} \tag{S8}$$

S5.2. Relative thermal sensitivity and temperature uncertainty

It is well established that the performance of a thermometer can be quantitatively expressed in terms of its relative thermal sensitivity (S_r), defined as:[14]

$$S_r = \frac{1}{\Delta}\left|\frac{\partial \Delta}{\partial T}\right| \tag{S9}$$

and in terms of its temperature uncertainty (δT):

$$\delta T = \frac{1}{S_r}\frac{\delta \Delta}{\Delta} \tag{S10}$$

where $\delta\Delta/\Delta$ is the relative uncertainty on the intensity ratio, that is estimated using:

$$\frac{\delta \Delta}{\Delta} = \sqrt{\left(\frac{\delta I}{I_{Tb}}\right)^2 + \left(\frac{\delta I}{I_{Eu}}\right)^2} \tag{S11}$$

where δI is the intensity fluctuation, obtained from the emission spectra (Figure S).

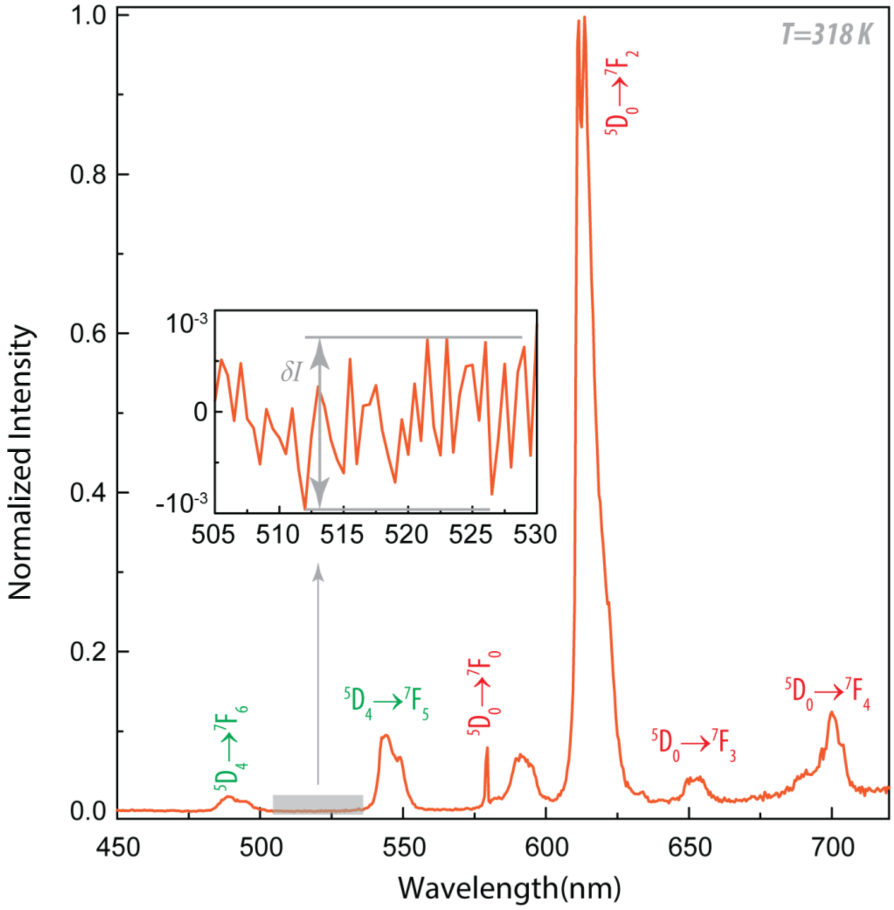

Figure S10. Emission spectrum (318 K) of the dU6EuTb excited at 254 nm, illustrating the procedure adopted to calculate δI.

Considering that Δ is described by Eq. S5, S_r is given by:

$$S_r = \frac{\Delta E}{k_B T^2} \times \frac{\alpha}{\exp\left(\frac{\Delta E}{k_B T}\right) + \alpha} \tag{S12}$$

and δT is given by:

$$\delta T = \frac{k_B T^2}{\Delta E} \times \frac{\alpha + \exp\left(\frac{\Delta E}{k_B T}\right)}{\alpha} \frac{\delta \Delta}{\Delta} \tag{S13}$$

S5.3 Repeatability

Repeatability was calculated by:

$$R = 1 - \frac{\max([\Delta_c - \Delta_i])}{\Delta_c} \quad (14)$$

where Δ_c is the thermometric parameter mean and Δ_i is the thermometric parameter calculated at each temperature.

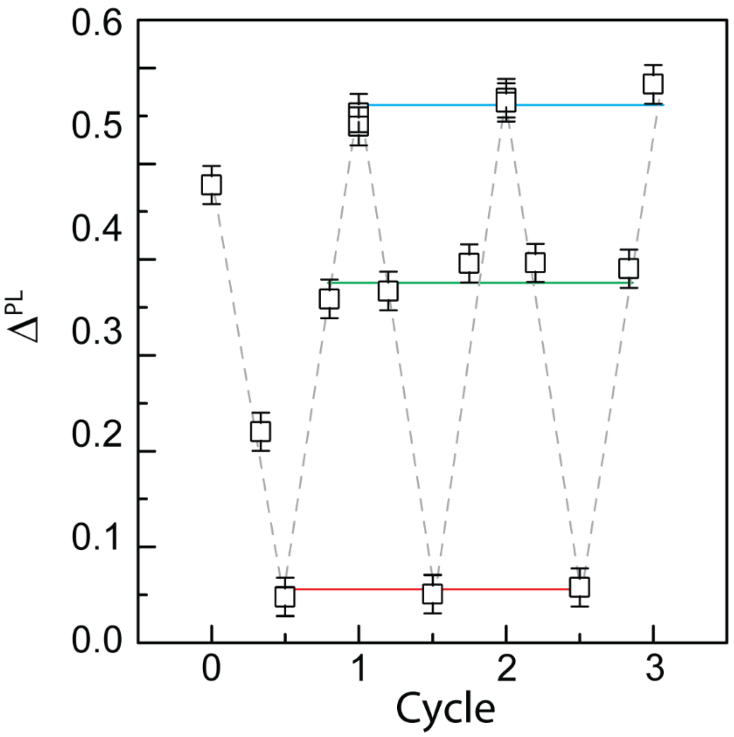

Figure S11. Repeatability of the thermometric parameter Δ^{PL} calculated for 3 complete temperature cycles between 283 and 317 K.

S5.4 Primary Thermometers

The attention on this topic was triggered by Souza *et al.*[15] that introduced the concept of predicting the temperature using the temperature dependence of the $^5D_0 \rightarrow {}^7F_4$ emission intensity when the $^5D_0$ level is directly excited from specific $^7F_1$ or $^7F_2$ Stark components thermally populated with respect to the $^7F_0$ non-degenerate ground state. The authors illustrate the principle using Y_2O_3:Eu^{3+} micro and nanocrystals with a relative thermal sensitivity up to 1.7% K^{-1}.

Later, Botas *et al.*[16] use Silicon nanoparticles (NPs) luminescence do develop a thermometer based

on the temperature dependent emission maximum shift. Upon 365 nm excitation the system display a single Gaussian profile ascribed to recombination of photogenerated electrons and holes located in the crystalline core of the NPs. The system is highly reproductible, however presenting a maximum relative thermal sensitivity of about 0.04 % K^{-1}.

More recently Balabhadra *et al.*[17] shown that the temperature dependence of any upconverting luminescent thermometer based on the transition intensity ratio originated in two thermally coupled emitting levels ($^2H_{11/2}$ and $^4S_{3/2}$) can be predicted using the Boltzmann equation for the intensity ratio of transitions originated in thermally coupled states. The authors used the Yb^{3+}/Er^{3+}-doped SrF_2 nanoparticles were used to show that the temperature calculated by the Boltzmann equation and that measured by a conventional thermometer in contact with the sample match within the error of both methods. A maximum relative thermal sensitivity of 1.21 % K^{-1} at 298.2 K was reported, in good agreement with the values listed for Er^{3+}-based thermometers in this temperature range. This work triggered the development of several systems that use the $^2H_{11/2}\rightarrow{}^4I_{15/2}$ and $^4S_{3/2}\rightarrow{}^4I_{15/2}$ transitions intensity to predict the temperature. The most recent works on applications of primary thermometers based on Er^{3+}-doped nanoparticles to electrochromic devices were reported by Martinez *et al.*.[18]

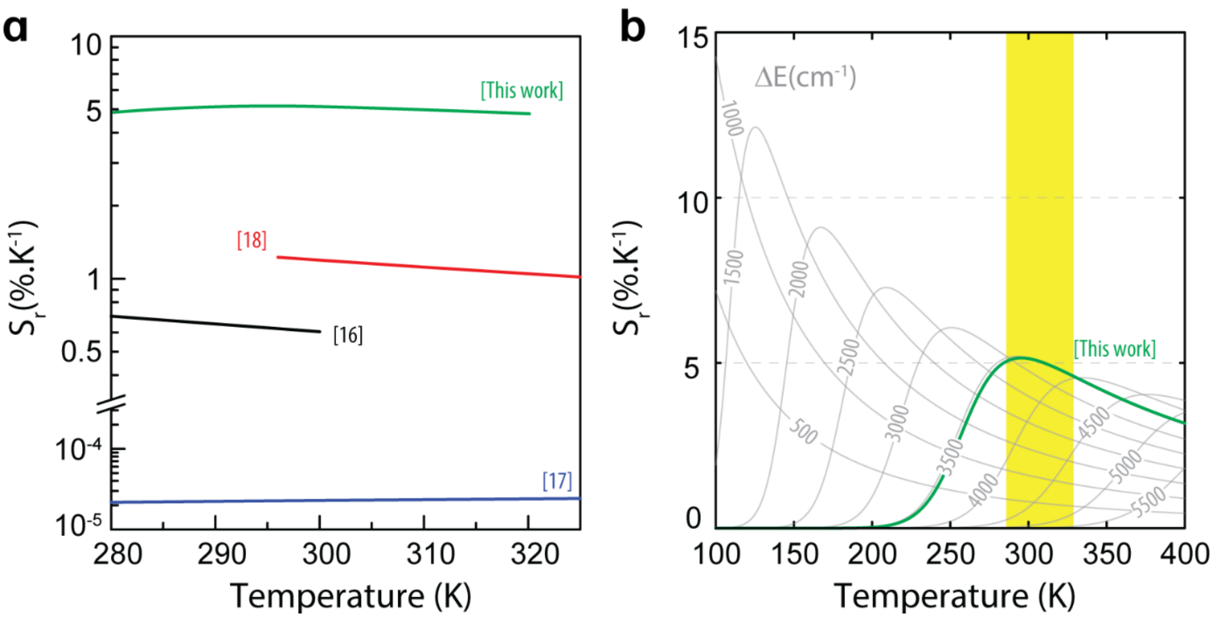

Figure S12. (a) Relative thermal sensitivity (S_r) of the primary luminescent thermometers. The S_m value reported here is the highest value reported so far for primary thermometer (a 4-fold increase with respect to the previous best value). **(b)** Predicted S_r curves according to Eq. S12, keeping the values of α and Δ_0 constant and changing the ΔE value between 500 and 5500 cm^{-1}. The shadowed area marks the temperature range in which the maximum sensitivity is attained by ΔE values

between 3500 and 4000 cm^{-1}. The curve resulting from the parameters of the thermometer reported here corresponds to the bold green line.

S5.5 QR code mobile App

A mobile application (App) devoted to QR code decoding and temperature reading was developed for an IOS based smartphone using programming language swift (version 5.0.1) and XCode compiler (version 10.2.1). Making use of the free UIKits avaliable for this language, namely *UIImage* class for image capture, processing, colour detection and quantification and *CIDectector* class for automatic shapes and pattern detection that assist in the QR code decoding, it was possible to structure the application in 4 main stages all connect and used in the presented order:

i) Image acquisition through the smartphone inbuilt rear camera or via stored photography in the smartphone's gallery (*UIImagePickerController* class);

ii) QR code detection (*CIDetectorTypeQRCode* class*)* and decoding (*CIQRCodeFeature* class) that when successfully made imply the presence of QR code and so, the luminescent material that provide the temperature sensing capability;

iii) Colour analyses and quantification using the mean RGB values of an area composed by 5% 5% pixels^2 of the image maximum width/height (*getPixelColor* - *UIImage* class extension and *getAreaColor*) centered at a user selected point for an intuitive interface (*UIGestureRecognizer* class);

iv) Based on the colour analyses using the reported values and methodology in the manuscript for temperature quantification.

The application is free and available for download at https://tinyurl.com/qr-luminescent.

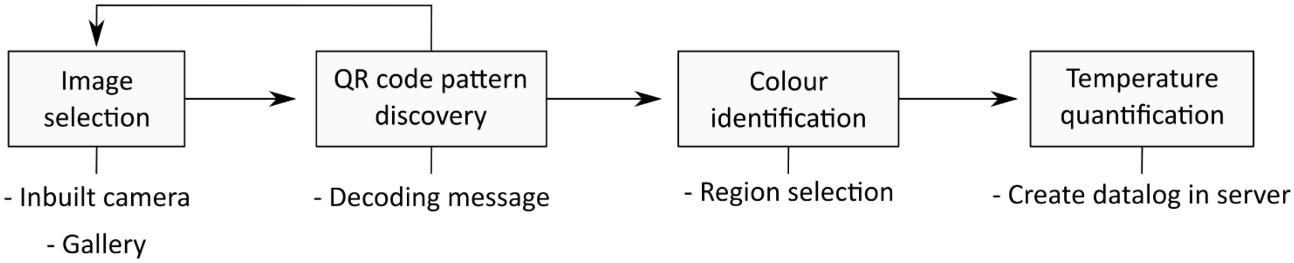

Figure S13. Schematic overview of the App structure.

References

[1] R. Ilmi, K. Iftikhar, J Coord Chem 2012, 65, 403.

[2] J. F. C. B. Ramalho, L. C. F. António, S. F. H. Correia, L. S. Fu, A. S. Pinho, C. D. S. Brites, L. D. Carlos, P. S. André, R. A. S. Ferreira, Optics & Laser Technology 2018, 101, 304.

[3] R. Ilmi, K. Iftikhar, J. Photoch. Photobio. A 2017, 333, 142.

[4] R. Ilmi, K. Iftikhar, Journal of Coordination Chemistry 2012, 65, 403.

[5] L. Carlos, V. de Zea Bermudez, R. Ferreira, L. Marques, M. Assunção, Chemistry of Materials 1999, 11, 581.

[6] C. Y. Liang, E. J. Schimitschek, J. A. Trias, Journal of Inorganic and Nuclear Chemistry 1970, 32, 811.

[7] C. D. S. Brites, M. C. Fuertes, P. C. Angelome, E. D. Martinez, P. P. Lima, G. J. A. A. Soler-Illia, L. D. Carlos, Nano Letters 2017, 17, 4746.

[8] V. V. Ovsyankin, Vol. 21 (Eds: A. A. Kaplyanskii, R. M. MacFarlane), Elsevier Science Publishers, Amsterdam 1987.

[9] L. D. Carlos, R. A. S. Ferreira, V. D. Bermudez, S. J. L. Ribeiro, Advanced Functional Materials 2001, 11, 111.

[10] L. D. Carlos, R. A. S. Ferreira, R. N. Pereira, M. Assunção, V. de Zea Bermudez, Journal of Physical Chemistry B 2004, 108, 14924.

[11] S. S. Nobre, P. P. Lima, L. Mafra, R. A. S. Ferreira, R. O. Freire, L. S. Fu, U. Pischel, V. de Zea Bermudez, O. L. Malta, L. D. Carlos, Journal of Physical Chemistry C 2007, 111, 3275.

[12] Y. Zheng, J. Lin, Y. Liang, Y. Yu, Y. Zhou, C. Guo, S. Wang, H. Zhang, Journal of Alloys and Compounds 2002, 336, 114.

[13] N. F. Mott, Proceedings of the Royal Society of London Series A-Mathematical and Physical Sciences 1938, 167, 0384; F. Seitz, Transactions of the Faraday Society 1939, 35, 0074.

[14] C. D. S. Brites, A. Millán, L. D. Carlos, in *Handbook on the Physics and Chemistry of Rare Earths*, Vol. 49 (Eds: J.-C. Bünzli, V. K. Pecharsky), Elsevier Science, B. V., Amsterdam 2016, 339; C. D. S. Brites, S. Balabhadra, L. D. Carlos, Advanced Optical Materials 2019, 7, 1801239.

[15] A. S. Souza, L. A. O. Nunes, I. G. N. Silva, F. A. M. Oliveira, L. L. da Luz, H. F. Brito, M. C. F. C. Felinto, R. A. S. Ferreira, S. A. Junior, L. D. Carlos, O. L. Malta, Nanoscale 2016, 8, 5327.

[16] A. M. P. Botas, C. D. S. Brites, J. Wu, U. Kortshagen, R. N. Pereira, L. D. Carlos, R. A. S. Ferreira, Particle and Particle System Characterization 2016, 33, 740.

[17] S. Balabhadra, M. L. Debasu, C. D. S. Brites, R. A. S. Ferreira, L. D. Carlos, Journal of Physical Chemistry C 2017, 121, 13962.

[18] E. D. Martínez, C. D. S. Brites, L. D. Carlos, A. F. García-Flores, R. R. Urbano, C. Rettori, Advanced Functional Materials 2019, 29, 1807758; E. D. Martínez, C. D. S. Brites, L. D. Carlos, R. R. Urbano, C. Rettori, Frontiers in Chemistry 2019, 7.
